# Effects of Preoperative High-intensity Training on Metabolic Flexibility

**DOI:** 10.1097/ALN.0000000000005487

**Published:** 2025-06-10

**Authors:** John Whittle, Zachary Healy, Jeroen Molinger, Stratton Barth, Anthony Molina, David MacLeod

**Affiliations:** 1 University College London, London, United Kingdom; 2 Duke University School of Medicine, Durham, North Carolina; 3 University of California-San Diego, San Diego, California

## To the Editor:

Cardiorespiratory fitness is a key predictor of postoperative outcomes, with low cardiorespiratory fitness linked to higher morbidity and mortality.^1^ Because low fitness is modifiable, prehabilitation is gaining popularity.^1^ However, the mechanisms linking cardiorespiratory fitness to surgical outcomes remain unclear. Higher cardiorespiratory fitness is associated with enhanced metabolic flexibility, the capacity to switch fuel use (fat *vs*. carbohydrate) to meet changing metabolic demands.^2^ In contrast, metabolic inflexibility, associated with systemic mitochondrial dysfunction, and common in individuals with low fitness, may impair immune responses and worsen postoperative recovery.^2–5^ Immune cells, particularly lymphocytes and monocytes, are highly sensitive to changes in the metabolic environment, and alterations in their metabolic flexibility can have profound effects on both their function and differentiation. Cardiopulmonary exercise testing is a standardized, noninvasive method widely used in the United Kingdom for preoperative evaluation. By measuring oxygen consumption and carbon dioxide production, cardiopulmonary exercise testing can estimate fat and carbohydrate oxidation, which can then be graphed as a “metabolic crossover” plot.^6^ At low-intensity exercise, fat utilization predominates, giving way to carbohydrate metabolism as intensity increases. Individuals with higher cardiorespiratory fitness show more efficient substrate switching, a trait associated with reduced postoperative morbidity. High-intensity interval training has been previously demonstrated to improve metabolic flexibility in nonsurgical populations. Up to 40% of the adult surgical population may present with metabolic syndrome, and significant physical deconditioning is common. Given the potential mechanistic importance of systemic metabolic flexibility in terms of perioperative outcomes, and its status as a modifiable risk factor, we hypothesized that metabolic flexibility, assessed through cardiopulmonary exercise testing and peripheral blood mononuclear cell respirometry, could be improved within typical prehabilitation timeframes in patients with cancer listed for major surgery.

In a prospective pilot interventional study (PREVENT, institutional review board Pro00090146 [Duke University, Durham, North Carolina]), 9 patients scheduled for major abdominal cancer surgery underwent cardiopulmonary exercise testing before and after a 6-week structured responsive exercise training (high-intensity interval training) program (10 patients were approached and 9 participated, all of whom completed the prescribed exercises). The regimen, three 30- to 40-min high-intensity interval training sessions a week, involved alternating 3-min moderate and 2-min high-intensity intervals, with intensity adjusted based on repeat cardiopulmonary exercise testing at week 3.^6,7^ Carbohydrate and fat utilization during exercise up to the ventilatory anerobic threshold were derived using standard stoichiometric equations and analyzed using the area under the receiver operating characteristics curve derived from fitted cubic regression curves.^8^ Blood samples collected before cardiopulmonary exercise testing allowed isolation of peripheral blood mononuclear cells (using Ficoll density gradient centrifugation), and respirometry (the measurement of cellular respiration, incorporating substrate utilization and oxygen consumption, to provide insight into mitochondrial function) was undertaken in triplicate on 3 × 10^5^ cells (see Supplemental Digital Content for detailed methods, https://links.lww.com/ALN/D953).

**Fig. 1.**
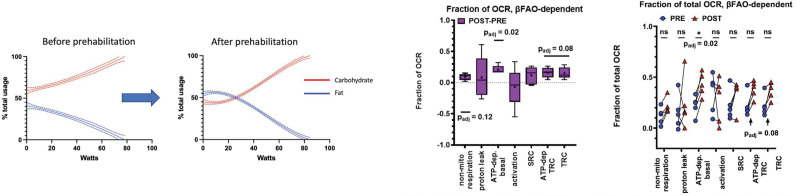
Fuel usage (carbohydrate, fat) during ramped cardiopulmonary exercise test (CPET) and peripheral blood mononuclear cell (PBMC) fraction of oxygen consumption rate (OCR) that is dependent on beta-oxidation of palmitate (long-chain fatty acid) (Seahorse XFp, Agilent Technologies, USA) before and after 6 weeks of structured high-intensity interval training. Fuel usage is expressed as percentage of total use at any given wattage (fat and carbohydrate). Activation, activation-induced (*i.e.*, CD3–CD28, simulating a standard immune response) respiration; ATP-dep basal, adenosine triphosphate–dependent basal respiration (excludes proton leak and nonmitochondrial oxygen consumption); βFAO, mitochondrial fatty acid beta oxidation; nonmitochondrial respiration, nonmitochondrial oxygen consumption; OCR, oxygen consumption rate; SRC, spare respiratory capacity; TRC, total respiratory capacity (*i.e.*, uncoupled with Bam15).

After prehabilitation, peak oxygen consumption increased from (mean ± SD) 12.4 ± 2.5 to 15.0 ± 4.1 ml · kg^–1^ · min^–1^ (*P* = 0.003), and ventilatory anaerobic threshold rose from 9.8 ± 1.9 to 11.7 ± 2.5 ml · kg^–1^ · min^–1^ (*P* = 0.01) (table 1). At rest, fat oxidation increased (0.45 ± 0.22 kcal/min to 0.76 ± 0.331 kcal/min; 95% CI, 0.09 to 0.6 kcal/min; *P* = 0.04), with a corresponding decrease in carbohydrate oxidation (1.60 ± 0.45 kcal/min to 1.29 ± 0.38 kcal/min; 95% CI, –3.1 to –0.11 kcal/min; *P* = 0.03; fig. 1). During exercise up to ventilatory anaerobic threshold, the area under the receiver operating characteristics curve for carbohydrate oxidation fell (7,247 ± 724.7 to 6,477 ± 388%/W; *P* = 0.05), while that for fat oxidation rose (1,923 ± 134.6 to 2,752 ± 165.1%/W; *P* = 0.05). Although overall peripheral blood mononuclear cell oxygen consumption and glycolysis parameters remained unchanged, long-chain fatty acid beta-oxidation dependency as a fraction of total oxygen consumption increased significantly (mean difference plus 21%; 95% CI, 0.13 to 0.29; adjusted *P* = 0.01), aligning with cardiopulmonary exercise testing substrate data (full results in the Supplemental Digital Content, https://links.lww.com/ALN/D953).

**Table 1.** Demographic, Cardiopulmonary Exercise Testing, and Peripheral Blood Mononuclear Cell Respirometry (Seahorse XF) Data before and after 6 Weeks of High-intensity Training–based Prehabilitation

	Before Prehabilitation	After Prehabilitation	95% CI	*P* Value	Adjusted *P* Value
Demographic data					
Male/female, No.	5/4				
Weight, kg	75.4 (54–122)	75.4 (56–121)		0.6	
BMI, kg/m^2^	29 (19–40)	29 (20–39)		> 0.5	
Age, yr	71 (65–81)				
ASA Physical Status	II (II–III)				
CPET metabolic cart data					
Peak V̇o_2_, ml · kg^–1^ · min^–1^	12.4 ± 2.5	15 ± 4	1.15 to 3.9	0.003	
VAT, ml · kg^–1^ · min^–1^	9.8 ± 1.9	11.7 ± 2.5	0.6 to 3.3	0.01	
MET	3.5 ± 0.76	4.3 ± 0.8	2.9 to 5.6	< 0.01	
Oxygen pulse, ml oxygen/beat	8.8 ± 3.5	10.8 ± 5.2	–0.56 to 4.6	0.1	
Ventilatory equivalents for carbon dioxide, V̇_E_V̇co_2_	31.52 ± 9.3	31.8 ± 9.3	–13.8 to 13.8	0.9	
Resting metabolic rate, kcal/h	85.3 ± 12	98.6 ± 10	–22 to 24.3	0.2	
Fraction βFAO-dependent OCR/total OCR, paired analysis					
Nonmitochondrial oxygen consumption	0.09 ± 0.05*		0.03 to 0.16		0.07
Proton leak	0.08 ± 0.34*		–0.33 to 0.5		0.85
ATP-dependent basal respiration	0.21 ± 0.07*		0.13 to 0.29		0.01
Activation-associated oxygen consumption	–0.07 ± 0.31*		–0.46 to 0.32		0.85
SRC	0.12 ± 0.14*		–0.06 to 0.29		0.35
ATP-dependent total respiratory capacity	0.16 ± 0.08*		0.06 to 0.26		0.07
Total respiratory capacity	0.15 ± 0.09*		0.04 to 0.27		0.07

Data are presented as median (interquartile range) or mean ± SD. *P* values are for paired *t* test. Oxygen consumption is in picomoles per minute per 325,000 cells. The blood draw for PBMC respirometry was conducted at rest before the first and last training session in the prehabilitation program. Seahorse XF, Agilent Technologies (USA).

* Value represents the difference (after – before prehabilitation).

ASA, American Society of Anesthesiologists; ATP, adenosine triphosphate; BMI, body mass index; CPET, cardiopulmonary exercise testing; FAO, fatty acid oxidation; MET, metabolic equivalents of task; PBMC, peripheral blood mononuclear cell; SRC, spare respiratory capacity; VAT, ventilatory anaerobic threshold: V̇o_2_, oxygen consumption.

In summary, a 6-week high-intensity interval training prehabilitation program was feasible in an elderly and significantly deconditioned population of surgical patients with cancer, significantly improving objectively measured cardiorespiratory fitness and enhancing systemic metabolic flexibility, including a shift toward greater long-chain fatty acid oxidation in peripheral blood mononuclear cells. This enhanced metabolic flexibility may be especially important in the early postoperative period when limited fuel availability coincides with increased metabolic demands. This adds to previous reports of improved metabolic flexibility in younger and fitter subjects after high-intensity interval training, demonstrating feasibility despite the challenges of delivering structured exercise in the busy time period before surgery. Given the known associations between low cardiorespiratory fitness, immune dysregulation, and postoperative infections, improving immune cell metabolic function through prehabilitation might reduce complications. Immune cells, particularly lymphocytes, require significant energy to mount an effective response to the surgical trauma.^3,4^ Shifts toward fatty acid oxidation are characteristic of T-cell metabolic reprogramming, which supports long-term immune function, promotes anti-inflammatory phenotypes, and reduces the metabolic cost of immune activation.^9^

Enhanced mitochondrial oxidative capacity in peripheral blood mononuclear cells, as evidenced by the increased adenosine triphosphate production from fatty acid oxidation, may improve the efficiency of immune responses, reduce inflammation, and mitigate the immune dysfunction often seen after major surgery. These findings are significant because impaired immune function, especially in older cancer patients, contributes to increased susceptibility to infections and other postoperative complications. Perhaps surprisingly, we did not observe significant changes in glycolytic metabolism after prehabilitation. Glycolysis is typically upregulated during acute immune activation, such as during infection or trauma, but our participants may not have exhibited such a response in the absence of these stimuli. Future studies incorporating postoperative immune profiling would help clarify the role of glycolysis in immune cell reprogramming after prehabilitation. While the direct impact of these metabolic changes on clinical outcomes remains to be determined, the enhancement of immune cell metabolic function through prehabilitation is an encouraging finding that warrants further investigation. These exploratory data are limited by a small sample size, absence of a control group, and the heterogeneous and changing nature of peripheral blood mononuclear cells during the early postoperative period. One outlier, who developed pneumonia during the study, notably influenced the results. Future studies should isolate specific immune cell populations and directly link changes in metabolic flexibility to clinical outcomes in the perioperative setting.

## Research Support

Funding for this study was provided by an International Anesthesia Research Society (San Francisco, California) Mentored Research Grant awarded to Dr. Whittle and by a grant from the National Institutes of Health (Bethesda, Maryland) to Dr. Healy (4R00-AI143927).

## Competing Interests

The authors declare no competing interests.

## Supplemental Digital Content

Supplemental Materials and Methods, https://links.lww.com/ALN/D953

## Supplementary Material


