# Supplementary material for: Effects of Preoperative High-intensity Training on Metabolic Flexibility
Source: Anesthesiology. 2025 Jun 10;143(1):217–20. doi: 10.1097/ALN.0000000000005487 (PMC12147750; doi:10.1097/ALN.0000000000005487)

Supplemental Materials and Methods.

Exercise Protocol:

The Exercise training protocol was taken from the EMPOWER study protocol [1]. This was an aerobic interval training program incorporating moderate and severe intensities performed supervised at Duke Cardiopulmonary rehabilitation center three times a week. Exercise intensity was derived from each individual CPET at week 0. Moderate intensity exercise was defined as being at a power output equivalent to 80% of the oxygen uptake at anaerobic threshold. Severe intensity was at a power output halfway between anaerobic threshold and peak VO_2. Each session consisted of a 5-minute warm-up and cool-down with unloaded pedalling. Exercise training intensities were responsive to CPET improvements and recalculated at week 3.

The first two training sessions consisted of 30 minutes of exercise, increasing to 40 minutes per session for all remaining sessions. In the first week of training, patients performed the interval training protocol for 20 mintues bracketed by the warm up and cool down. The interval phase included four bouts of moderate (3 minutes) to severe intensity (2 minutes). After week one, the time of the interval session increased to 30 minutes, consisting of 6 bouts of moderate (3 minutes) to severe intensity (2 minutes) intervals.

The program was conducted on computer controlled electromagnetically braked cycle ergometers (Optibike Ergoselect 200; Ergoline, GmbH, Bitz, Germany). Heart rate was continually recorded from R-R interval (Polar, Warwick, UK). The program was preloaded onto a chip card that executed the individualised exercise program automaticlly on the cycle ergometer. The Study timeline is illustrated in supplemental figure 1.

PBMC Isolation. Cell preservation and culture was performed as described[1]. For study subjects, peripheral whole blood was obtained from Duke IRB-approved (Pro00090146) anonymous donors using ACD vacutainer tubes (BD Biosciences), and PBMCs were isolated using Ficoll density centrifugation (GE HealthCare). PBMCs were counted and viably cryopreserved in LN_2 vapor (10% DMSO, 90% heat-inactivated FBS). Thawing of cells was performed in RPMI-1640 media containing 10% heat-inactivated FBS (Gibco) and 1x penicillin-streptomycin-glutamine (Gibco) at 37°C and 5% CO_2. Cell counts and viability were checked using the ThermoFisher Countess II cell counter. Cells were rested for two hours prior to being plated for Seahorse experiments. We did not perform a substrate limitation incubation prior to the assay as our intention was to study the metabolic state of the cells that most closely resembled *in vivo* conditions. Cells were plated at 3×10^5 cells/well in PDL-coated plates for the assay, with at least duplicates run for each condition.

Agilent Seahorse XF Cellular Metabolism Assay. All assays were performed using the Seahorse XFp platform as described[2]. The following reagents were purchased from Agilent including: RPMI media, pH 7.4; glucose (1M stock); L-glutamine (200 mM stock); sodium pyruvate (100 mM stock); XF Palmitate-BSA Fatty Acid Oxidation substrate kit; PDL-coated plates. The XF Palmitate-BSA Fatty Acid Oxidation kit

contains palmitate conjugated to BSA, and as such tests only metabolism of long-chain fatty acids (LCFA) and does not account metabolism of medium- or short-chain fatty acids which may be present in the cytoplasm at the time of the assay. We chose to analyse long-chain fatty acid metabolism as LFCAs are the predominant form in which FAs are stored by the body for use as an energy source. The following reagents were purchased from Cayman Chemical: oligomycin, BAM15, rotenone, antimycin A, etomoxir, and 2-deoxy-D-glucose. Media for the assay was prepared the day of the assay and contained: Agilent Seahorse RPMI, pH 7.4, 5 mM glucose, 2 mM glutamine, 1 mM sodium pyruvate, 0.5 mM L-carnitine, and 30 µL/well of Agilent Palmitate-BSA stock (concentration not provided by manufacturer). Antibodies against human CD3 and CD28 (for T cell stimulation) were purchased from Biolegend. To test the dependence of energy production on beta-oxidation of long-chain fatty acids, etomoxir (4 µM) was used. These inhibitors were added to the cells during the 1-hour de-gassing processing prior to the start of the assay and were present throughout. Dependency of oxygen consumption (OCR), and similarly, extracellular acidification (ECAR) were determined as follows (substituting ECAR for OCR where appropriate):

$$OCR_{TOTAL} = OCR_{\beta FAO-dependent} + OCR_{\beta FAO-dependent}$$

$$OCR_{TOTAL} = OCR_{Solvent+Palmitate}$$

$$OCR_{\beta FAO-independent} = OCR_{\ Etomoxir+Palmitate}$$

$$OCR_{\beta FAO-dependent} = OCR_{Solvent+Palmitate} - OCR_{Etomoxir+Palmitate}$$

$$Fraction\ of\ OCR_{\beta FAO-depedent} = \frac{OCR_{\beta FAO-depedent}}{OCR_{TOTAL}}$$

Concentrations of compounds for the MitoStress Assay were as follows: injection (1) CD3-CD28, 1 and 5 µM respectively, final concentrations); (2) oligomycin, 2.5 µM; (3) BAM15, 20 µM – note this higher concentration was required in the presence of BSA; (4) rotenone, 0.5 µM, and antimycin-A, 0.5 µM. All data were normalized to total DNA content in the well, which was measured using the ThermoFisher QuBit high-sensitivity DNA kit. Data were analyzed in Prism GraphPad (v10) using multiple paired t-tests for pre-determined metabolic parameters (shown in Figure X). Individual variance values were used for each group, and the Holm-Sidak method was used to calculate adjusted P values for multiple comparisons (significance, $*p_{adj} < 0.05$; ns, not significant). CI (95%) intervals were calculated for each variable as well.

Supplemental results: Peripheral Blood Mononuclear Cell Respirometry (Seahorse XF ®) before and after 6 weeks of High Intensity Interval Training based prehabilitation. Oxygen consumption is in pmol/min/325,000 cells. The blood draw for PBMC respirometry was conducted at rest before the first and last training session in the prehabilitation program

TOTAL OCR, Unpaired	Before Prehabilitation				After Prehabilitation				
Variable	Mean	StDev	Lower Limit, 95% CI	Upper Limit, 95% CI	Mean	StDev	Lower Limit, 95% CI	Upper Limit, 95% CI	Adjusted p value
Non-mitochondrial O_2 consumption	24.59	2.68	21.26	27.92	23.03	2.02	20.51	25.54	24.59
Proton leak	11.31	3.14	7.41	15.21	10.40	5.13	4.03	16.76	11.31
ATP-dependent basal respiration	47.27	3.75	42.61	51.92	49.48	12.04	34.53	64.43	47.27
Activation-associated O_2 consumption	28.66	8.08	18.63	38.69	15.16	6.31	7.33	22.99	28.66
Spare respiratory capacity (SRC)	104.79	9.87	92.53	117.04	87.58	52.50	22.39	152.77	104.79
ATP-dependent total respiratory capacity	152.37	8.59	141.70	163.03	137.81	54.25	70.44	205.17	152.37
Total respiratory capacity	166.08	13.37	149.49	182.68	148.27	57.72	76.61	219.94	166.08

βFAO-dependent OCR, Unpaired	Pre-intervention	Post-intervention	

Variable	Mean	StDev	Lower Limit, 95% CI	Upper Limit, 95% CI	Mean	StDev	Lower Limit, 95% CI	Upper Limit, 95% CI	Adjusted p value
Non-mitochondrial O_2 consumption	3.01	2.22	0.25	5.77	5.01	2.28	2.19	7.84	3.01
Proton leak	3.05	4.45	-2.47	8.57	3.24	4.53	-2.38	8.87	3.05
ATP-dependent basal respiration	10.42	4.68	4.60	16.23	21.88	9.92	9.56	34.20	10.42
Activation-associated O_2 consumption	10.38	7.80	0.70	20.07	4.24	3.50	-0.10	8.59	10.38
Spare respiratory capacity (SRC)	22.55	13.33	6.00	39.10	32.35	24.36	2.10	62.59	22.55
ATP-dependent total respiratory capacity	33.28	15.69	13.80	52.77	54.97	29.43	18.43	91.51	33.28
Total respiratory capacity	36.36	16.23	16.21	56.51	58.20	33.07	17.14	99.26	36.36

**Fraction βFAO-dependent OCR/total OCR, Unpaired**	Pre-intervention	Post-intervention	

Variable	Mean	StDev	Lower Limit, 95% CI	Upper Limit, 95% CI	Mean	StDev	Lower Limit, 95% CI	Upper Limit, 95% CI	Adjusted p value
Non-mitochondrial O_2 consumption	0.12	0.08	0.02	0.22	0.21	0.08	0.12	0.31	0.12
Proton leak	0.15	0.17	-0.05	0.36	0.24	0.25	-0.07	0.55	0.15
ATP-dependent basal respiration	0.22	0.10	0.10	0.34	0.43	0.11	0.29	0.57	0.22
Activation-associated O_2 consumption	0.34	0.19	0.10	0.58	0.27	0.22	-0.01	0.55	0.34
Spare respiratory capacity (SRC)	0.22	0.15	0.04	0.40	0.34	0.15	0.16	0.52	0.22
ATP-dependent total respiratory capacity	0.22	0.11	0.08	0.36	0.38	0.08	0.28	0.49	0.22
Total respiratory capacity	0.22	0.10	0.09	0.35	0.37	0.09	0.27	0.48	0.22

1. Healy ZR, Weinhold KJ, Murdoch DM. Transcriptional Profiling of CD8+ CMV-Specific T Cell Functional Subsets Obtained Using a Modified Method for Isolating High-Quality RNA From Fixed and Permeabilized Cells. Front Immunol. 2020 Sep 2;11:1859. doi: 10.3389/fimmu.2020.01859
2. Suliman HB, Healy Z, Zobi F, Kraft BD, Welty-Wolf K, Smith J, Barkauskas C, Piantadosi CA. Nuclear respiratory factor-1 negatively regulates TGF-β1 and attenuates pulmonary fibrosis. iScience. 2021 Dec 1;25(1):103535. doi: 10.1016/j.isci.2021.103535. PMID: 34977500; PMCID: PMC8683592.
3. Romero N, Rogers G, Neilson A, Dranka B. Quantifying Cellular ATP Production Rate using Agilent Seahorse XF Technology. White Paper. Agilent

Technologies, Inc., Lexington, MA, USA. Last Accessed November 2024 at: https://www.agilent.com/cs/library/whitepaper/public/whitepaper-quantify-atp-production-rate-cell-analysis-5991-9303en-agilent.pdf

4. Agilent Seahorse XF CO_2 Contribution Factor Protocol. S7888-10011, Agilent Technologies, Inc.2020.

Supplemental Figure 1: PREVENT Study Timeline

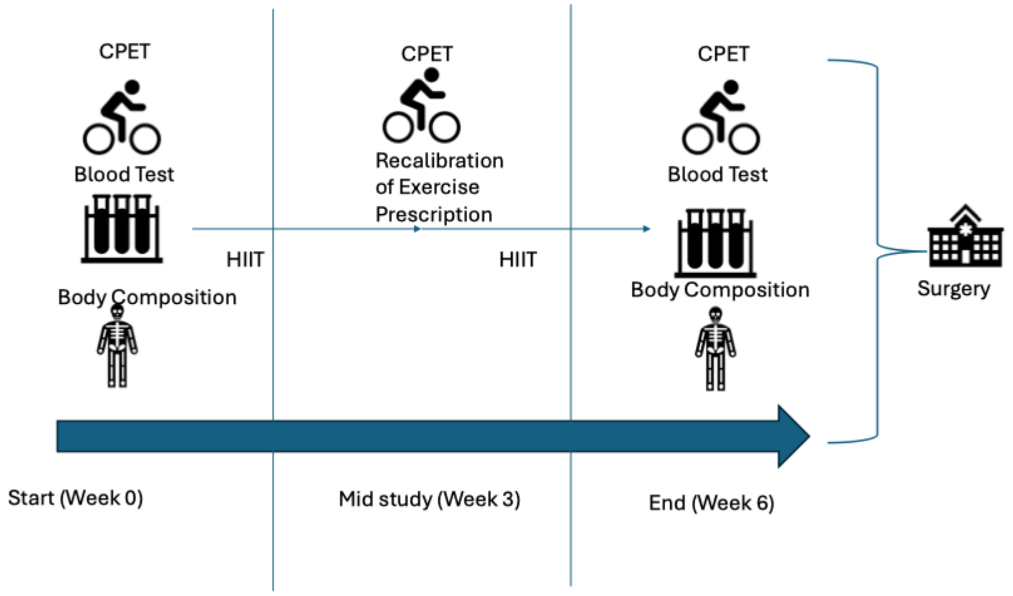

Supplement: Supplementary file 1 [file aln-143-217-s001.pdf]
